# Evaluation of the epidemiological behavior of mortality due to COVID-19 in Brazil: A time series study

**DOI:** 10.1371/journal.pone.0256169

**Published:** 2021-08-12

**Authors:** Ketyllem Tayanne da Silva Costa, Thiffany Nayara Bento de Morais, Dayane Caroliny Pereira Justino, Fábia Barbosa de Andrade

**Affiliations:** Department of Nurse, Federal University of Rio Grande do Norte, Rio Grande do Norte, Brazil; University Magna Graecia of Catanzaro, ITALY

## Abstract

The World Health Organization declared, at the end of 2019, a pandemic caused by SARS-CoV-2, a virus that causes Coronavirus Disease—COVID-19. Currently, Brazil has become the epicenter of the disease, registering approximately 345 thousand deaths. Thus, the study has scientific relevance in health surveillance as it identifies, quantifies and monitors the main behavioral patterns of the mortality rate due to COVID-19, in Brazil and in their respective regions. In this context, the study aims to assess the epidemiological behavior of mortality due to COVID-19 in Brazil: a time series study, referring to the year 2020. This is an ecological time series study, constructed using secondary data. The research was carried out in Brazil, having COVID-19 deaths as the dependent variable that occurred between the 12th and 53rd Epidemiological Week of 2020. The independent variable will be the epidemiological weeks. The data on deaths by COVID-19 were extracted in February 2021, on the Civil Registry Transparency Portal. The cleaning of the database and the information were treated in the Microsoft Excel® Software and, for statistical analysis, the JoinPoint software, version 4.7.0.0 was used. It was observed that Brazil presents an upward curve between the 12th and 19th SE, when it reaches saturation at the peak of mortality, which remains until the 35th SE and, subsequently, a downward curve was identified until the 47th SE, period in the which curve turns back up.

## Introduction

On March 11, 2021, the World Health Organization—WHO (2021) declared a pandemic caused by SARS-CoV-2, a virus that originated in Wuhan, Hubei province, China, at the end of 2020, and that causes Coronavirus Disease—COVID-19. The emerging virus has a respiratory character and can cause mild symptoms or worsen for critical conditions, with massive alveolar damage and respiratory failure [1–3].

The pandemic has advanced rapidly, reaching about 192 countries. Worldwide, 2,694,915 deaths have been recorded by COVID-19. Currently, Brazil has become the epicenter of the disease, registering 287,489 deaths, second only to the United States of America (539,868), on March 19, 2021 [4]. Even with emergency and definitive approval for the use of vaccines against COVID-19, Brazil continues to record a record of deaths each day. According to researchers from the Oswaldo Cruz Foundation—Fiocruz (2021), the country experiences the greatest sanitary collapse in its history [5].

The Centers for Diseases Control and Prevention—CDC (1992) conceptualizes public health surveillance as the set of actions for the collection, analysis, interpretation and continuous and systematic dissemination of data on the health of a population [6]. In the meantime, in the face of the collapse of public health caused by the virus, it is important to understand the main events that have a high impact on the behavior associated with the mortality rate due to COVID-19.

Thus, the study has scientific relevance in health surveillance as it identifies, quantifies and monitors the main behavioral patterns of the mortality rate due to COVID-19, in Brazil and in their respective regions. Thus, contributing to observe changes in the patterns of occurrence of the virus, evaluating preventive and control measures, consequently influencing the improvement of the planning of health programs [7].

In this context, the study aims to evaluate the epidemiological behavior of mortality due to COVID-19 in Brazil: a time series study, referring to the year 2020.

## Materials and methods

This is an ecological time series study, built using secondary data. The research was carried out in Brazil, having COVID-19 deaths as the dependent variable that occurred between the 12th and 53rd Epidemiological Week of 2020, which corresponds to the period from March 15, 2020 to January 2, 2021, according to the regions of Brazil.

Epidemiological Week is an international standard that groups deaths and other epidemiological events. This division allows the data for each period to be analyzed and compared among themselves, between countries, in addition to several years, being a year composed of 52 or 53 epidemiological weeks [8].

The regions are formed by states, which have similar characteristics to each other, therefore, to obtain a better visualization of the national reflex, the data were categorized by regions and, thus, compared with the Brazilian standard.

The dependent variable selected for the construction of the study was the mortality rate (M) caused by COVID-19. To calculate the rate, the absolute number of deaths per COVID-19 was divided by the number of inhabitants of Brazil and multiplied by 1,000,000. The Brazilian population, according to the Brazilian Institute of Geography and Statistics, in the 2010 census is made up of about 190,732,694 inhabitants. The independent variable will be the epidemiological weeks (1) [9].


$$M=\frac{Mortality\;by\;COVID-19}{Population}X\;1,000,000$$ (1)

The data on deaths by COVID-19 were extracted in February 2021, on the Civil Registry Transparency Portal, and can be viewed on the site <https://transparencia.registrocivil.org.br/especial-covid>. The platform records, daily, deaths in Brazil from the actual detection of death information.

After the collection, the database was cleaned and the information was treated in the Microsoft Excel® Software. Then, to obtain the statistical analysis, the software JoinPoint, version 4.7.0.0, provided by the National Cancer Institute of the United States (http://surveillance.cancer.gov/joinpoint/), whose access is free.

JoinPoint uses linear regression trends to obtain junction points, in time series. The output consists of the Annual Percentage Change (APC) and the Average Annual Percentage Change (AAPC), which indicate significant variations in the rate analyzed. The tests are based on the significance calculated from the Monte Carlo Permutation method. The results obtained in JoinPoint are presented in epidemic curves, in order to build a visualization of the behavior caused by COVID-19.

Therefore, the epidemic curve is a graphical representation of the behavior of a given disease over a period of time. The organization takes place on an axis of coordinates, the horizontal being represented by time, and the vertical, the frequencies. The disease may present an asymmetric curve, composed of ascending curves, exemplifying the growth phase and the speed at which the frequency of the disease occurred, the maximum point, which represents the peak, and the descending curve, which is the representation of the exhaustion of the epidemic, through natural measures or control measures imposed [7].

The data used for the construction of the present study were secondary and extracted from the public domain database, so as not to use personal data, and therefore, not needing appreciation by the Ethics and Research Committee.

## Results

Table 1 summarizes the results obtained by linear regression of deaths caused by COVID-19 in Brazil and regions, according to SE. In the analysis of the country, it was possible to observe 4 JoinPoint, however, when the regions were observed, there was a discrepancy between 3 JoinPoint in the South and 6 JoinPoint in the Northeast. In the meantime, it is clear that Brazil has two APCs with statistical significance, the first between the 12th and 19th SE and the other between the 35th and 47th SE. This behavior is similar in the Northeast and Southeast regions, while in the Midwest and South, the significance is shown later, whereas the North region presents earlier statistical significance. In the regression, however, it exhibits significant AAPC both in Brazil and in the regions, with the North being the region with the greatest inconsistency in mortality and the Southeast being the most stable region, in addition, it was also observed that there was a pattern in the occurrence of jumps in the 47th epidemiological week.

**Table 1:** Mortality rate due to COVID-19 in Brazil and regions in the period from 12ª to 53ª epidemiological week. Brazil, 2020.

Local	Jump Point	Joinpoint^1^	Period	APC^2^	Lower	Upper	AAPC^3^	Lower	Upper
Brazil	16	19, 35, 47, 50	12 to 19	49.5*	33.2	67.8	7.3*	2.8	12.0
			19 to 35	0.0	-1.4	1.4			
			35 to 47	-9.9*	-12.7	-7.1			
			47 to 50	54.9	-6.6	157.1			
			50 to 53	0.5	-14.4	17.9			
North	16	18, 21, 25, 28, 47	12 to 17	174.5*	80.0	318.7	12.4*	5.5	19.7
			17 to 20	26.7	-1.7	63.4			
			20 to 25	-22.0*	-28.8	-14.5			
			25 to 28	18.3	-24.8	86.2			
			28 to 47	-8.3*	-9.8	-6.8			
			47 to 53	26.6*	16.2	38.0			
Northeast	16	19, 22, 25, 28, 47, 50	12 to 19	90.4*	61.8	124.1	9.2*	2.1	16.9
			19 to 31	-3.6*	-5.7	-1.3			
			31 to 47	-10.2*	-12.4	-8.0			
			47 to 50	54.2	-33.3	256.7			
			50 to 53	-0.7	-21.6	25.8			
Midwest	16	32, 41, 47, 50	12 to 32	23.4*	21.1	25.8	8.6*	6.3	10.9
			32 to 41	-7.7*	-10.2	-5.2			
			41 to 47	-22.5*	-29.9	-14.3			
			47 to 53	26.6*	17.1	36.9			
Southeast	16	18, 36, 47, 50	12 to 19	40.3*	27.8	54.0	6.1*	2.1	10.4
			19 to 37	-1.4*	-2.6	-0.3			
			37 to 47	-9.7*	-13.3	-5.9			
			47 to 50	55.3	-2.8	148.2			
			50 to 53	1.1	-12.9	17.3			
South	16	32, 47, 50	12 to 32	18.1*	16.5	19.7	8.7*	6.5	11.0
			32 to 47	-7.6*	-8.7	-6.4			
			47 to 50	62.4*	25.4	110.2			
			50 to 53	-5.3	-13.5	3.7			

^1^by epidemiological week.

^2^annual percentage change.

^3^average annual percentage change.

*p-value <0.05.

**Fig 1:**
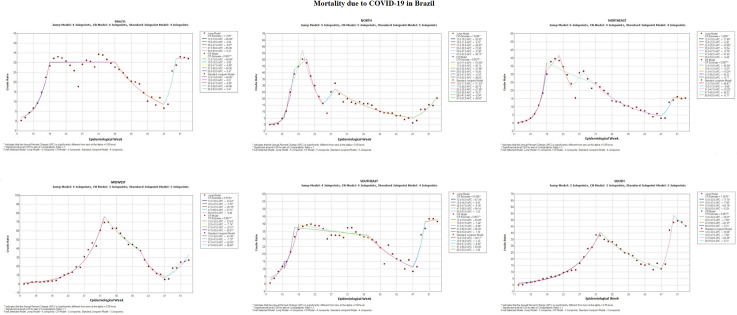
Mortality rate due to COVID-19 in Brazil and regions in the period from 12ª to 53ª epidemiological week. Brazil, 2020.

Fig 1 shows that the polynomial models of the studied period. In the first part, which concerns the evolution of the mortality rate in Brazil, there was a drastic increase in the mortality rate due to COVID-19 in the period between the 12th to the 19th SE. The same behavior can be seen in the North, Northeast and Southeast regions. However, in the Midwest and South regions, the curve rose gradually until the 32nd SE. It is still possible to observe the occurrence of stability in the evolution of the mortality rate from the 19th to the 35th SE, in Brazil. However, it is important to highlight that the only Brazilian region that also had this behavior was the southeast region, which reveals the strength of the occurrence of deaths in this region. In this Fig 1, it is worth noting that the North and Northeast regions showed different patterns in the evolution of the mortality rate, when compared to the other regions, showing a peak between SE 19 and 23 (57/million and 41/million people, respectively), followed by a sharp reduction and, subsequently, a further rise between the 25th and 31st SE. In the 32nd, there was a peak in the mortality rate in the Midwest region (73/million people), with the involution of the mortality rate from the 35th SE to the 47th, where it rose again until the 53 SE.

It is worth noting that there was a pattern of a second wave of rise in the mortality rate from the 47th SE onwards in all regions. During this period, the South reaches its peak (54/million people) in the 50th SE. In the last JoinPoint, from the 50th to the 53rd SE, the graph of Brazil showed a trend of stability at the peak, as well as in the Northeast region. The Southeast, on the other hand, continued to grow until reaching its peak (43/million people) in the 53rd SE. The North and Center-West regions experienced an increase, while the South showed a reduction in the same period.

## Discussion

The results presented start from the 12th Epidemiological Week (SE), when all regions of the country started to present a significant mortality rate due to COVID-19. Brazil recorded its first death from COVID-19 on March 17, 2020, being the victim of the male gender, 62 years old and resident of São Paulo, the state with the highest population density, according to IBGE (2010), thus being able to have influenced directly in the curve of the southeast region [10].

However, only in the 13th SE, the state of São Paulo established mandatory quarantine, so that this late decision reflected an accelerated spread of the virus in the weeks following the decree, making it one of the states with the worst statistics [11].

As a consequence of the decree, population adherence to quarantine reached a percentile of 59% of the population of São Paulo, being even higher than the percentile of the country, which was 52.3% in the 14th SE [12, 13]. Still, it is possible to identify that isolation obtained better rates on weekends, because on working days the flow of residents remained high, even with the operation of only essential services.

It is also worth mentioning that, in Brazil, the high demand and the unpreparedness for such a proportion of the severity that the virus caused, generated a low supply of beds, reduced professional quantity to meet the need, in addition to the scarce individual protection material and physical structure that would support the existing demand added to the demand generated by the pandemic. Thus, it caused the public and private health system to collapse, leading to high mortality rates in several regions, as shown in Fig 1.

The first case in Brazil was only registered about 3 months after the outbreak in Wuhan, China. Unlike Brazil, in China a quick response was shown to contain the outbreak of COVID-19 [11]. From the epidemiological data, the Chinese government observed that the most developed locations and that had a greater installed capacity of health resources had lower mortality rates. In this perspective, the need arose to make health resources available to other more precarious regions of China, including the epicenter of the disease. Thus, the mortality rate is possibly associated with the installed health capacity, so it is essential to invest in the preparation of sites even at the beginning or before the arrival of the virus [14].

Therefore, it is observed that the lack of preparation to meet the needs of the pandemic in Brazil started since the lack of planning in the period when the virus had not arrived in the country and this lack of health management corroborated the occurrence of a sudden increase in the mortality rate in the country, in the period between the 12th and 19th SE, as can be seen in Fig 1. At that moment, the country registered 28,734 hospitalizations for Severe Acute Respiratory Syndrome (SRAG), whereas, in the same period of 2019, the number was 1,700 hospitalizations [15].

Paim (2013) defines that, when there is an issue of concern with the need for public service to the population, the provision of services must occur, fulfilling the spontaneous demand of the organized offer [16]. Therefore, in view of the pandemic experienced in the world due to the coronavirus, there was an intense demand in hospital care, thus, when this demand was not met, a marked deterioration in public health is noticeable, becoming even clearer in the mortality rate. In Brazil, even with the data stabilization, the number of deaths prevailed with high rates for 16 consecutive epidemiological weeks.

Decree nº 10.316, of April 7, 2020, regulates the so-called Emergency Aid, a social protection measure adopted by Brazil to minimize the economic impact caused by the SARS-CoV-2 pandemic [17]. The financial aid was intended for people with low income and / or who lost their income during quarantine, as self-employed and unemployed. The unemployment rate in the country reached a record 13.9% in 2020, however, the Midwest and South were the regions with the lowest unemployment rates, consequently, received the least amount of Emergency Aid, in addition to having the lowest illiteracy rate in Brazil [18–20]. In the meantime, it can be seen in the results obtained that both regions showed a delay in the mortality rate due to COVID-19, as the population has a greater understanding of the disease and more easily adheres to the preventive protocols proposed by WHO and, therefore, In this way, they present a growth pattern that is distinct from the other regions.

The World Health Organization (1973) determines that epidemiology is constituted from three main objectives, among them to provide essential data for the planning, execution and evaluation of the actions of prevention, control and treatment of diseases, as well as to establish priorities [21]. In this perspective, Matus (1987) builds the theory of situational strategic planning (PES), which has a public character, in order to assist in the gestational planning of public health, with the participation of various actors, through conflict and cooperation situations [22].

Thus, in an attempt to curb and subsequently mitigate the mortality rate, the Ministry of Health, through Ordinance No. 1,514, of June 15, 2020, decreed the implementation of Temporary Health Units for Hospital Assistance, known as Hospitals of Campaign, being totally focused on assisting patients in the scope of the emergency due to the pandemic of COVID-19, this containment measure being taken at the 25th SE [23]. Considering the time for the construction and operation of hospitals, a drop in the mortality curve in Brazil was observed only in the 35th SE.

However, the mortality rate rose again in the 47th SE, in Brazil, as well as in all regions of the country, coinciding with the first round of the municipal electoral process. In this perspective, it is important to note that the political campaign started in the 40th SE, causing a greater flow of people, which can be observed by the rate of adherence to social isolation, this being the lowest index described throughout the year 2020, around 35% [13]. It is known that the virus incubation period is an average of 5.2 days, in addition, the patient can transmit the virus for 7 days after incubation [24].

Therefore, it was observed that social isolation has drastically reduced due to the culture of mass agglomerations during political events, given that this situation was identified in all Brazilian states, especially in the interior cities. Still, these events were used by political parties as a tool to omit the pandemic context and minimize the severity of the virus, through demonstrations, rallies, home visits, among others. It is noteworthy that many candidates for the election did not encourage the use of a mask, alcohol 70º INPM and social distance, since the candidates themselves did not follow the WHO recommendations [25]. These agglomerative focuses were permitted by the Superior Electoral Court, through Law No. 9.504/1997, art. 38, §§ 9 and 11, between September 27 and November 14 [26]. In this perspective, it is worth noting that the epidemiological curve, which followed a downward pattern, rose again on November 15 (47th SE), noting that the agglomerations that occurred in this period contributed to the increase in deaths from COVID-19 in Brazil, as shows Fig 1.

According to data obtained by the Oswaldo Cruz Foundation—Fiocruz (2020), the percentage of the flow of people at home, after an increase from March, fell in May, with a tendency to regress, obtaining numbers below 20% [27]. This behavior can be seen in several Brazilian states, as shown in the data in the month of May, where the percentile of stores and recreation, workplaces and transport stations obtained a significant increase in the flow of people, when compared to the beginning of the quarantine, all above 20%. The data presented reflect the greater flexibility in preventive measures as of May, when Brazil is saturated in the epidemiological curve, as can be seen in Fig 1.

Thus, the limitations of the study are linked to the lack of accessibility of the data, since constant changes were observed in the pattern of presentation of information contained in epidemiological sources in Brazil. In addition, due to the recent emergence of the virus, the scarcity of studies on the subject in the Brazilian scenario is noticeable, making it difficult to analyze certain parameters for a broad understanding of the impact of the disease in the country.

## Conclusions

Given the above, the study brought data on the behavior of deaths by COVID-19 in Brazil and its regions, in the year 2020, being possible to observe that the Brazilian regions started to present statistical significance in the mortality rate after the 12th Week Epidemiological (SE). Thus, it is noteworthy that Brazil presents an upward curve between the 12th and 19th SE, when it reaches saturation at the peak of mortality, which remains until the 35th SE and, subsequently, a downward curve was identified until the 47th SE, period in which the curve ascends again.

The aforementioned regional variations are associated with the behavior of Brazilian society in relation to preventive measures, as well as the country’s public health structure and the decisions adopted by government officials at the federal, state and municipal levels. In this context, it is noteworthy that the regions exhibit singular, but similar behaviors at times, such as the increase in the mortality rate in the 47th SE, a period that coincides with the municipal electoral campaign and this requires the health services to be properly prepared to receive of the new cases of COVID-19.
